# Supplementary material for: Factors associated with the uptake of Intermittent Preventive Treatment (IPTp-SP) for malaria in pregnancy: Further analysis of the 2018 Nigeria Demographic and Health Survey
Source: PLOS Glob Public Health. 2023 Feb 1;3(2):e0000771. doi: 10.1371/journal.pgph.0000771 (PMC10021516; doi:10.1371/journal.pgph.0000771)
Supplement: S1 Table — This is the extension of Table 4 with the interaction term. (DOCX) [file pgph.0000771.s001.docx]

**S1 Table.**

**Interaction term between Household wealth Index and Spouse’s Educational level**

| **Household Wealth Index** | **Spouse’s Educational levels – aOR^1^ (95% CI)** | | | **Interaction p-value** |
| --- | --- | --- | --- | --- |
|  | **Primary** | **Secondary** | **Higher** | **0.045^a^** |
| **Poorest** | 1.00 | 1.00 | 1.00 |  |
| **Poorer** | 1.00 (0.56–1.79) | 1.15 (0.72 – 1.82) | 0.89 (0.25 – 3.15) |  |
| **Middle** | 1.06 (0.53–2.10) | **2.03 (1.24 – 3.30) **** | 0.87 (0.24 – 3.10) |  |
| **Richer** | 1.22 (0.55–2.67) | 1.61 (0.83 – 3.10) | 0.72 (0.20 – 2.64) |  |
| **Richest** | 1.29 (0.41– 4.03) | **3.16 (1.11 – 9.01) *** | 1.32 (0.29 – 6.00) |  |
| *^a^p-value – overall p-value of the interaction term*  *95% CI – 95% Confidence Intervals, aOR^1^ – Adjusted Odds ratio*  *aOR^1^ was adjusted for all factors presented in Table 4 (uptake of at least one dose of IPTp-SP)*  **p<0.05, **p<0.01 (Actual p-value *p= 0.032; **p =0.005)* | | | | |
