# Supplementary material for: Factors associated with the uptake of Intermittent Preventive Treatment (IPTp-SP) for malaria in pregnancy: Further analysis of the 2018 Nigeria Demographic and Health Survey
Source: PLOS Glob Public Health. 2023 Feb 1;3(2):e0000771. doi: 10.1371/journal.pgph.0000771 (PMC10021516; doi:10.1371/journal.pgph.0000771)
Supplement: S2 Table — This is the initial model without the interaction term for the uptake of at least one SP dose. (DOCX) [file pgph.0000771.s002.docx]

**S2 Table. The model without the interaction term for the uptake of at least IPTp-SP dose**

| **Factors** | **Total N (%)** | **Crude ORs (95% CI)**  **Uptake of at least one dose** | **P-value** | **Adjusted ORs (95%CI)**  **Uptake of at least one dose** | **P-value** |
| --- | --- | --- | --- | --- | --- |
| **Socio-demographic Factors** | | | | | |
| **Age in years** |  |  | **0.011** |  | **0.008** |
| 15-24 | 3,842 (30.2) | 1.00 |  | 1.00 |  |
| 25-34 | 6,242 (49.0) | 1.17 (1.06 - 1.29) | 0.003 | 1.10 (0.93 - 1.32) | 0.245 |
| 35+ | 2,657 (19.3) | 1.12 (0.98 - 1.28) | 0.100 | 1.42 (1.12 - 1.80) | 0.004 |
| **Region** |  |  | **<0.001** |  | **<0.001** |
| NorthCentral | 1,770 (13.9) | 1.00 |  | 1.00 |  |
| North East | 2,339 (18.4) | 1.42 (1.17 - 1.73) | <0.001 | 2.07 (1.65 - 2.61) | <0.001 |
| North West | 4,639 (36.4) | 1.08 (0.88 - 1.31) | 0.465 | 2.58 (2.03 - 3.29) | <0.001 |
| South East | 1,263 (9.9) | 2. 96 (2.36 - 3.71) | <0.001 | 1.39 (1.06 - 1.81) | 0.016 |
| South South | 1,126 (8.8) | 2.22 (1.817 - 2.700) | <0.001 | 1.66 (1.26 – 2.16) | <0.001 |
| South West | 1,606 (12.6) | 1.34 (1.08 - 1.66) | 0.007 | 0.52 (0.40 - 0.66) | <0.001 |
| **Residential areas** | |  | **<0.001** |  | **0.066** |
| Urban | 4,853 (38.1) | 1.00 |  | 1.00 |  |
| Rural | 7,888 (61.9) | 0.52 (0.45 - 0.60) | <0.001 | 1.17 (0.99 - 1.37) | 0.066 |
| **Highest Educational Level** | |  | **<0.001** |  | **0.919** |
| No Education | 5,766 (45.3) | 1.00 |  | 1.00 |  |
| Primary | 1,856 (14.6) | 2.01 (1.74 - 2.33) | <0.001 | 0.98 (0.80 - 1.19) | 0.829 |
| Secondary | 4,050 (31.8) | 2.75 (2.41 - 3.14) | <0.001 | 0.94 (0.76 - 1.15) | 0.520 |
| Higher | 1,070 (8.4) | 4.371 (3.53 - 5.42) | <0.001 | 0.91 (0.67 - 1.24) | 0.554 |
| **Household wealth Index** | |  | **<0.001** |  | **<0.001** |
| Poorest | 2,763 (21.7) | 1.00 |  | 1.00 |  |
| Poorer | 2,933 (23.0) | 1.29 (1.10 - 1.51) | 0.001 | 0.93 (0.76 - 1.13) | 0.464 |
| Middle | 2,636 (20.7) | 2.31 (1.97 - 2.72) | <0.001 | 1.32 (1.06 - 1.64) | 0.014 |
| Richer | 2,358 (18.5) | 2.98 (2.49 - 3.57) | <0.001 | 1.34 (1.03 - 1.74) | 0.028 |
| Richest | 2,052 (16.1) | 4.58 (3.75 - 5.61) | <0.001 | 1.82 (1.35 - 2.45) | <0.001 |
| **Employment Status** | |  | **<0.001** |  | **0.780** |
| Not Employed | 3,915 (30.7) | 1.00 |  | 1.00 |  |
| Employed | 8,827 (69.3) | 1.46 (1.31 - 1.62) | <0.001 | 1.02 (0.88 - 1.18) | 0.780 |
| **Spouse’s Education Level** | |  | **<0.001** |  | **<0.001** |
| No Education | 4,568 (35.6) | 1.00 |  | 1.00 |  |
| Primary | 1,624 (12.7) | 2.09 (1.77 - 2.46) | <0.001 | 1.12 (0.87 - 1.44) | 0.375 |
| Secondary | 4,116 (32.7) | 3.07 (2.69 - 3.51) | <0.001 | 1.58 (1.28 - 1.94) | <0.001 |
| Higher | 1,872 (14.7) | 4.54 (3.80 - 5.42) | <0.001 | 1.46 (1.11 - 1.93) | 0.007 |
| **Pregnancy-related factors** | | | | | |
| **Frequency of ANC visits** | |  | **<0.001** |  | **<0.001** |
| <4 ANC Visits | 2,350 (24.7) | 1.00 |  | 1.00 |  |
| ≥ 4 ANC Visits | 7,151 (75.3) | 1.50 (1.31 - 1.72) | <0.001 | 1.60 (1.35 - 1.89) | <0.001 |
| **Timing of First ANC Initiation** | |  | **0.001** |  | **0.143** |
| 1st Trimester | 2,273 (23.6) | 1.00 |  | 1.00 |  |
| 2nd Trimester | 6,003 (62.3) | 0.96 (0.83 - 1.12) | 0.631 | 0.98 (0.83 - 1.16) | 0.855 |
| 3rd Trimester | 1,365 (14.2) | 0.71 (0.59 - 0.86) | <0.001 | 0.82 (0.65 - 1.03) | 0.094 |
| Number of children born |  |  |  |  |  |
| <4 children | 6692 (52.7) | 1.00 |  | 1.00 |  |
| 4+ children | 6049 (47.5) | 0.78 (0.71 – 0.85) | <0.001 | 0.92 (0.78 – 1.09) | 0.353 |
| **Knowledge of malaria-related factors** | | | | | |
| **Media Exposure** |  |  | **<0.001** |  | **0.142** |
| No | 11,822 (92.8) | 1.00 |  | 1.00 |  |
| Yes | 919 (7.2) | 2.69 (2.14 - 3.37) | <0.001 | 1.22 (0.94 - 1.58) | 0.142 |
| **Health Insurance Subscription** | |  | **0.001** |  | **0.076** |
| No | 12,484 (98.0) | 1.00 |  | 1.00 |  |
| Yes | 257 (2.0) | 2.51 (1.47 - 4.28) | 0.001 | 1.57 (0.95 - 2.58) | 0.076 |
| **Belief in Effectiveness of IPTp-SP** | |  | **<0.001** |  | **<0.001** |
| Low belief | 200 (1.6) | 1.00 |  | 1.00 |  |
| Average belief | 504 (4.0) | 0.69 (0.47 - 1.03) | 0.072 | 0.51 (0.26 - 1.01) | 0.055 |
| High belief | 12,038 (94.5) | 2.47 (1.81 - 3.38) | <0.001 | 1.51 (0.90 - 2.52) | 0.111 |
| **Belief about Malaria Consequences** | |  | **<0.001** |  | **0.009** |
| Low belief | 1,614 (12.7) | 1.00 |  | 1.00 |  |
| Average belief | 3,869 (30.4) | 1.38 (1.17 - 1.62) | <0.001 | 1.34 (1.06 - 1.70) | 0.015 |
| High belief | 7,258 (57.0) | 1.49 (1.29 - 1.72) | <0.001 | 1.35 (1.12 - 1.64) | 0.002 |
| *uptake of at least one dose of IPTp-SP implies uptake of at least one dose of IPTp-SP; ANC – Antenatal care*  *p-values – overall p-values for each exposure variable in the model, CI – Confidence Intervals*  *OR – Odds ratios, crude OR (from Bivariate analysis) Adjusted OR (from Multivariable analysis)*  ***Goodness-of-fit of the model = F (9, 1262) = 0.800; p = 0.616*** | | | | | |
