# Supplementary material for: Factors associated with the uptake of Intermittent Preventive Treatment (IPTp-SP) for malaria in pregnancy: Further analysis of the 2018 Nigeria Demographic and Health Survey
Source: PLOS Glob Public Health. 2023 Feb 1;3(2):e0000771. doi: 10.1371/journal.pgph.0000771 (PMC10021516; doi:10.1371/journal.pgph.0000771)
Supplement: S1 Text — (DOCX) [file pgph.0000771.s003.docx]

**“Table A in S1 Text”**

**The raw data (scores) for the belief about malaria morbidity before categorisation**

| **Malaria morbidity score** | **Frequency (N)** | **Percentage (%)** |
| --- | --- | --- |
| 1.00 | 111 | 0.9 |
| 1.25 | 13 | 0.1 |
| 1.50 | 1,593 | 12.6 |
| 1.75 | 115 | 0.9 |
| 2.00 | 3,595 | 28.5 |
| 2.25 | 289 | 2.3 |
| 2.50 | 5,464 | 43.3 |
| 2.75 | 93 | 0.7 |
| 3.00 | 1,351 | 10.7 |
| **Total** | **12,624** | **100.0** |

**“Table B in S1 Text”**

**The raw data (scores) for the belief about IPTp effectiveness before categorisation**

| **IPTp effectiveness score** | **Frequency (N)** | **Percentage (%)** |
| --- | --- | --- |
| 1.00 | 214 | 1.7 |
| 1.50 | 10 | 0.1 |
| 2.00 | 503 | 4.0 |
| 2.50 | 51 | 0.4 |
| 3.00 | 11,846 | 93.8 |
| **Total** | **12,624** | **100.0** |
